# Alterations in implantable cardioverter defibrillator lead parameters following left ventricular assist device implantation

**DOI:** 10.1016/j.jhlto.2025.100350

**Published:** 2025-07-21

**Authors:** David Gittess, David J. King, Steven Brady, Ang Li, Yi Guo, Sara Geiger, Mustafa M. Ahmed, Alex M. Parker, Ramil Goel

**Affiliations:** a Division of Internal Medicine, University of Florida, Gainesville, FL; b Health Outcomes & Biomedical Informatics, University of Florida, Gainesville, FL; c Division of Cardiovascular Medicine, University of Florida, Gainesville, FL

**Keywords:** Electrophysiology, Advanced heart failure, Implantable cardioverter defibrillator (ICD), Left ventricular assist device (LVAD), Right ventricular lead disfunction

## Abstract

**Background:**

Left ventricular assist devices (LVADs) are increasingly used in the management of advanced heart failure. The majority of these patients have pre-existing implantable cardioverter defibrillators (ICDs). The proximity between the LVAD inflow cannula and right ventricular (RV) defibrillation lead raises the potential for disruption of ICD function.

**Methods:**

This is a retrospective analysis of 95 patients with ICDs at a single tertiary care center who underwent LVAD implantation and who met inclusion criteria. The primary outcome was changes in the pre-operative and post-operative transvenous ICD RV lead parameters. These changes were stratified by the age of the RV lead and analyzed via a paired t-test. The secondary outcome was disruption to the ICD requiring an intervention.

**Results:**

LVAD implantation was associated with significant decreases in sensed amplitude (p < 0.01) and high voltage impedance (p < 0.01) and an increase in capture threshold (p = 0.017). When stratified by age of the RV lead, patients with leads older than two years had similar trends in all parameters. However, RV leads that were two years old or younger only showed a significant change in high voltage impedance (p < 0.01). Mechanical disruption of the ICD related to the surgery was infrequent but significant.

**Conclusion:**

Because LVAD implantation is capable of impacting ICD function and causing mechanical disruption, close monitoring should be paid to the ICD in the peri-operative period including obtaining a full interrogation.

## Introduction

Contemporary treatment of chronic systolic heart failure includes goal-directed medical therapy, and implantable medical devices as indicated. These devices include implantable cardioverter defibrillators (ICDs) and, for those individuals who progress to advanced heart failure, left ventricular assist devices (LVADs). While ICD utilization in the general heart failure population is associated with clear benefits,^1^, ^2^, ^3^ the overall benefit for ICD implantation in the LVAD population is unclear.^4^, ^5^, ^6^

Most advanced heart failure patients who require LVADs usually have pre-existing ICDs that are often kept in place. The placement of the LVAD in the left ventricular apex has the potential to affect the RV lead which is traditionally positioned in the right ventricular apex. The impact of LVAD implantation on ICD function has not been fully characterized. Previous analyses have shown alterations in ICD parameters, most prominently changes in the sensed amplitude and impedance.^7^, ^8^, ^9^, ^10^, ^11^, ^12^, ^13^, ^14^ These studies have also documented infrequent cases for ICD disruption including lead fractures and dislodgment. In addition, many case reports have documented electromagnetic interference between LVADs and ICDs across numerous device manufacturers that have impacted ICD parameters or interfered with ICD function significantly enough to warrant ICD replacement.^15^, ^16^, ^17^, ^18^

This study represents one of the largest attempts to identify trends in ICD disruption with particular focus on alterations in ICD parameters, electrical failure of the ICD, and mechanical damage during LVAD implantation, stratified by age of the right ventricular (RV) lead. This information can be used to guide patient management post-operatively and to highlight common complications to ICD function that will allow for more prompt intervention.

## Materials and methods

### Study design

This study comprised a single-center retrospective chart review of an advanced heart failure treatment center at the University of Florida (Gainesville, FL, USA) between June 1, 2011 and December 31, 2021. Patients were included if they met the following criteria: aged greater than 18 years old with advanced heart failure who had a pre-existing transvenous ICD and subsequently underwent implantation of an LVAD at the University of Florida. Patients without an ICD prior to LVAD implantation and those with an existing ICD but without a device interrogation recorded within one year before and after LVAD implantation were excluded. The primary outcome was changes in ICD parameters. The secondary outcome was disruption of the ICD components requiring an intervention like device reprogramming.

ICD interrogations were reviewed independently by two authors (DG and DK) and any disagreements were resolved by consensus or review by a senior electrophysiologist (RG) to assess RV lead sensed amplitude (millivolts), impedance (ohms), capture threshold (volts), pulse width (ms), and high voltage impedance (ohms). The interrogations taken closest to VAD implantation were used. Adverse events such as lead fractures were identified in operative, hospital, and clinic notes. Demographic information was similarly recorded from hospital files. This study was approved by an Institutional Review Board at the University of Florida (IRB202102992).

### Device characteristics

In patients that met the inclusion criteria, all device parameters were collected in the analysis. The interrogated ICDs were manufactured by Medtronic (Dublin, Ireland), Biotronik (Berlin, Germany), and Boston Scientific (Marlborough, Massachusetts, USA). St. Jude Medical (formerly Sunnyvale, California, USA) is now owned by Abbott Cardiovascular (Plymouth, Minnesota, USA), so these devices have been designated with both names. LVAD models include the HeartMate 2 (HM2) and HeartMate 3 (HM3) by Abbott Cardiovascular and the Heartware (HVAD) by Medtronic.

### Statistical analysis

Demographic and device information were evaluated with descriptive statistics. Associations and changes in ICD parameters were analyzed with a paired t-test and stratified by lead age of less than or equal to two years and greater than two years.

## Results

**Figure 1:**
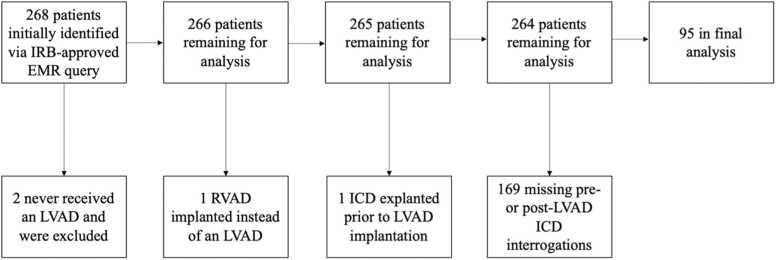
Flow diagram.

There were 268 patients in the sample. Of these, two were worked up for an LVAD but never received it. One patient’s ICD was explanted prior to receiving his LVAD, and he had another placed after surgery. One patient underwent implant of a temporary RVAD and was excluded. A further 169 patients did not have complete data, principally complete ICD interrogations within one year before and after the VAD implant. This left a final sample of 95 patients. A flow diagram of subject exclusion is recorded in Figure 1. Demographic data is recorded in Table 1.

**Table 1:** Patient Demographics (n=95)

Average Age at Time of LVAD Implant		58.4±11.8 Years Old
Sex	Male	72 (75.8%)
	Female	23 (24.2%)
Ethnicity	White	57 (60.0%)
	African American	30 (31.6%)
	Hispanic/Latino	4 (4.2%)
	Asian	2 (2.1%)
	Other/did not specify	2 (2.1%)
Average BMI (kg/m^2^)	29.1	
Average ejection fraction	13.6%	
Cardiomyopathy	Nonischemic	60 (63.2%)
	Ischemic	35 (36.8%)
History of hypertension	Yes	61 (64.2%)
	No	34 (35.8%)
History of diabetes	Yes	51 (53.7%)
	No	44 (46.3%)
History of CAD	Yes	58 (61.1%)
	No	37 (38.9%)
History of atrial fibrillation or atrial flutter	Yes	50 (52.6%)
	No	45 (47.4%)
History of COPD	Yes	43 (45.3%)
	No	52 (54.7%)
Tobacco use	Non-smoker	39 (41.1%)
	Former smoker	41 (43.2%)
	Active smoker	15 (15.8%)
Alcohol	Abstinent	0 (0.0%)
	Clinically insignificant*	90 (94.7%)
	Formerly clinically significant alcohol use*	5 (5.3%)
Illicit substance use	Prior use	6 (marijuana, cocaine)
	Active use	1 (marijuana)
Received transplant		15 (15.8%)

*Clinically significant alcohol use defined as greater than 2 drinks per night or 14 drinks per week if male and one drink per night or 7 drinks per week if female.

Most LVAD patients had either a HM2 (46.3%) or HM3 (33.7%), and the remaining 20.0% had an HVAD implanted. One patient had a pump thrombosis necessitating the sole LVAD exchange in the sample, from an HVAD to an HM3 device. The most common ICD and RV lead were both manufactured by Medtronic (50.5% and 46.3%, respectively), followed by Boston Scientific/Guidant (23.2% for ICD and 26.3% for RV lead), St. Jude Medical/Abbott Cardiovascular (22.1% for both ICD and RV lead), and finally Biotronik (4.2% for ICD and 5.3% for RV lead). Most ICDs were biventricular (52.6%) or single chamber devices (29.5%). The average age of the RV lead prior to LVAD implant was 4.4 ± 3.7 years. Device information is recorded in Table 2. On average, the device interrogations were recorded 78.9 days (median 50 days) after LVAD implant. By device, the interrogations were recorded at an average of 96.5 days (median 66 days) after implant for HVADs, 78.1 days (median 23 days) for HM2 devices, and 69.5 days (median 49 days) for HM3 devices.

**Table 2:** Device Information

	Device Model	# of Patients
LVAD manufacturer (first)	Heartmate 2	44 (46.3%)
	Heartmate 3	32 (33.7%)
	Heartware	19 (20.0%)
LVAD manufacturer (second)	Heartmate 3	1 device
ICD manufacturer	Medtronic	48 (50.5%)
	Boston Scientific/Guidant	22 (23.2%)
	St. Jude Medical/Abbott Cardiovascular	21 (22.1%)
	Biotronik	4 (4.2%)
ICD device class	Single chamber	28 (29.5%)
	Dual chamber	17 (17.9%)
	Biventricular	50 (52.6%)
RV lead manufacturer	Medtronic	44 (46.3%)
	Boston Scientific/Guidant	25 (26.3%)
	St. Jude Medical/Abbott Cardiovascular	21 (22.1%)
	Biotronik	5 (5.3%)
Average RV lead age prior to LVAD		4.4±3.7 years
Average (median) days to interrogation	All devices	78.9 (50)
	Heartmate 2	78.1 (23)
	Heartmate 3	69.5 (49)
	Heartware	96.5 (66)

Initial analysis of the change in ICD parameters is noted in Table 3. This data is significant for decreases in the sensed amplitude (p < 0.01) and high voltage impedance (p < 0.01), as well as an increase in the capture threshold (p = 0.017) following LVAD implantation. When stratified by age of the RV lead, there was a significant decrease in the high voltage impedance (p < 0.01) for leads implanted within 2 years of LVAD surgery (n=33). For leads older than two years (n=62), there were significant decreases in the sensed amplitude and high voltage impedance (both p < 0.01) and an increase in the capture threshold (p = 0.026). The data was also tested for associations with different LVAD devices (Table 4). Implantation of all three LVAD devices in the study was associated with a decrease in high voltage impedance (P < 0.01). In addition, the HM3 was associated with a decrease in sensed amplitude (p = 0.019).

**Table 3:** Overall Changes in Parameters and Changes in Parameters Stratified by Lead Age

Overall Changes in Parameters (n=95)			p-value
Sensing	Pre-LVAD	10.70±5.15	<0.01
	Post-LVAD	9.09±5.87	
Impedance	Pre-LVAD	461.69±120.40	0.857
	Post-LVAD	463.36±151.60	
Capture Threshold	Pre-LVAD	1.00±0.57	0.017
	Post-LVAD	1.18±0.81	
Pulse width	Pre-LVAD	0.46±0.14	0.206
	Post-LVAD	0.49±0.19	
High Voltage Impedance	Pre-LVAD	53.21±15.28	<0.01
	Post-LVAD	46.2±17.25	
*Changes in parameters stratified for RV lead age less or equal to than 2 years (n=33)*			
Sensing	Pre-LVAD	10.89±4.87	0.537
	Post-LVAD	10.43±6.74	
Impedance	Pre-LVAD	458.27±79.84	0.333
	Post-LVAD	445.24±100.87	
Capture Threshold	Pre-LVAD	0.96±0.67	0.405
	Post-LVAD	1.03±0.83	
Pulse width	Pre-LVAD	0.46±0.12	0.211
	Post-LVAD	0.48±0.17	
High Voltage Impedance	Pre-LVAD	56.82±15.41	<0.01
	Post-LVAD	50.85±16.73	
*Changes in parameters stratified for RV lead age greater than 2 years (n=62)*			
Sensing	Pre-LVAD	10.60±5.33	<0.01
	Post-LVAD	8.38±5.27	
Impedance	Pre-LVAD	463.52±137.78	0.438
	Post-LVAD	474.0±172.64	
Capture Threshold	Pre-LVAD	1.03±0.51	0.026
	Post-LVAD	1.25±0.78	
Pulse width	Pre-LVAD	0.46±0.15	0.374
	Post-LVAD	0.49±0.19	
High Voltage Impedance	Pre-LVAD	51.29±14.98	<0.01
	Post-LVAD	43.73±17.14

**Table 4:** Associations Between ICD Lead Parameters and Type of LVAD

LVAD model	Pre-LVAD sensing	Post-LVAD sensing	p value
HM2	10.87±4.89	10.08±5.81	0.167
HM3	11.09±5.63	8.44±6.62	0.019
HVAD	9.64±5.03	7.89±4.37	0.116
	Pre-LVAD impedance	Post-LVAD impedance	p value
HM2	461.48±103.50	462.32±122.25	0.94
HM3	462.41±123.84	479.06±186.07	0.434
HVAD	461.00±153.95	439.32±153.73	0.132
	Pre-LVAD capture threshold	Post-LVAD capture threshold	p value
HM2	1.01±0.64	1.08±0.80	0.31
HM3	0.97±0.52	1.31±0.95	0.077
HVAD	1.05±0.50	1.18±0.56	0.096
	Pre-LVAD pulse width	Post-LVAD pulse width	p value
HM2	0.45±0.08	0.47±0.13	0.21
HM3	0.45±0.11	0.50±0.24	0.174
HVAD	0.53±0.26	0.53±0.20	1
	Pre-LVAD high voltage impedance	Post-LVAD high voltage impedance	p value
HM2	49.82±13.90	42.95±16.44	<0.01
HM3	56.66±15.20	49.06±18.56	<0.01
HVAD	55.26±17.49	48.89±16.32	<0.01

Among the main study sample, 12 experienced some form of device disruption (Table 5). Mechanical disruptions included lead fractures (2), dislodgement (1), intra-operative lead transection (1), and other general disruptions manifesting as device failure. Often, failure would be referred to as “lead malfunction” without other specification. For four subjects, a device malfunction was noted, though no device damage was identified. This most commonly manifested as inappropriate shocks (2). For five patients, the affected device component was turned off, preventing future interrogations. Device complications were excluded from this list if they occurred prior to surgery or over one year after surgery, or if they underwent intervention for routine causes like a generator exchange for low battery.

**Table 5:** Mechanical and Electrical Dysfunction of ICDs After LVAD Surgery

Combined Disruptions (n=8)		
Type of Mechanical Disruption	Type of Electrical Disruption (If Noted)	Intervention (If Any)
Damage to lead (RV)	Decreased impedance (RV)	Lead revision (RV)
Intra-operative: atrial lead cut	n/a	removal of lead, not re-implanted
Lead externalization (RV)	n/a	lost to follow up, with generator replacement 2 years later, followed by Micra 6 months after generator replacement
Intra-operative: lead dislodgement (LV)	n/a	LV lead turned off
RV lead malfunction	Inappropriate shocks and elevated high voltage impedance	Lead revision (RV)
LV lead malfunction	Non-capture (epicardial LV) in patient with VT	LV lead turned off
RA lead malfunction	no RA sensing	n/a
Lead fracture vs dislodgement (RV)	Increased impedance and pacing threshold, failure to capture at a low threshold	ICD therapies turned off, lead revision (RV) two months later
Electrical disruptions only (n=4)		

Type of electrical disruption	Intervention (if any)	
Inappropriate shock for atrial fibrillation with RVR	Turned off low frequency attenuation filter	
Inappropriate shock	IV amiodarone instead of PO dofetilide due to concerns for poor PO absorption	
Decreased sensing and increased pacing threshold (RA, RV)	LV pacing activated	
No explanation given	LV lead turned off	

## Discussion

### LVAD implantation can impact the structure and function of ICDs

Our study is one of the largest to date and demonstrates that the implantation of an LVAD significantly impacts ICD parameters and can cause damage to the device itself. The ICD parameters most likely to be affected are the sensed amplitude, capture threshold, and high voltage impedance.

Several mechanisms have been proposed to explain how LVAD implantation can affect ICD leads. The most immediately apparent is mechanical disruption, whether non-catastrophic damage, damage compromising the lead function, or damage to the contact points of the lead and myocardium during surgery. Further, this event prompts post-surgical myocardial remodeling that can distort the anatomy of the heart and by extension the location of the lead relative to the rest of the myocardium.^7^ In addition, several case reports have attributed RV dysfunction to electromagnetic interference from the LVAD.^16^, ^18^ Hu et al took an interesting approach of measuring ICD parameters at several stages intraoperatively and only noted a change to sensed amplitude and impedance as bypass was being weaned.^11^ Although they acknowledged that their small sample size precluded generalizable conclusions, they suggested that LV unloading and septal shift to the left can account for parameter changes, especially considering that imaging before and after surgery did not show changes in lead positioning. Further, they noted that changes in the sensed amplitude threshold improved over the weeks after surgery, possibly also owing to post-operative ventricular remodeling. In comparison to their study, our study did not follow device parameters serially over many time points but rather only at one post-operative time at an average of 78.9 (median 50) days after surgery. The time to interrogation can confound the interpretation of these results, as unrelated damage to an ICD can accrue in the time between LVAD implant and interrogation. In addition to these causes, loss of capture or alterations in the capture threshold or high voltage impedance can have organic explanations. These include acidosis or orientation of the lead relative to the myocardium, which can be altered by long-term post-LVAD myocardial remodeling.^19^, ^20^, ^21^

The clinical relevance of these changes is unclear. Alterations in sensed amplitude could increase the likelihood of inappropriate shocks. However numerous patients in the study had decreases in sensed amplitude without this complication. Given the importance of ICDs in terminating dangerous arrhythmias, a better sense of the impact of these lead changes is important. The ability to clearly identify and respond to these arrhythmias is the core function of the ICD and is of importance in LVAD patients given their underlying substrate and the increased risk of ventricular arrhythmias in the post-operative period.^22^, ^23^ Data on the mortality associated with ventricular arrhythmias in LVAD patients is contradictory; however, at longer durations of follow up, the data suggest – though do not definitively demonstrate – increased mortality in patients with post-LVAD ventricular arrhythmias.^24^, ^25^

This data also provides insight into the impact that the type of LVAD can have on changes in ICD parameters. Notably, the high voltage impedance showed significant decreases across all three devices, suggesting that the change is related to mechanical offloading of the LV with LVADs in general more so than an interaction with one specific LVAD device. The only other significant change was a decrease in sensed amplitude among HM3 devices. This is somewhat surprising; while all LVADs can provide a benefit to reverse myocardial remodeling, an analysis comparing axial and centrifugal flow devices found that the remodeling benefit was comparable across all three devices in terms of LV recovery as measured by change in ejection fraction.^26^ Moreover, a comparison of the devices themselves does not offer an obvious explanation. The HM3 and HVAD are both centrifugal flow devices which are intrapericardial with similar implant techniques and positioning; however, they did not share the decrease in sensed amplitude. The HM2, meanwhile, would be expected to have greater changes in ICD parameters because the axial flow necessitates higher RPMs, and the anchoring of the device displaces the LV apex.^26^, ^27^ This results in a more conical form of the LV apex and decreased LV end diastolic diameter.^27^ It is ultimately unclear why only the HM3 exhibited an additional change in parameters.

Curiously, younger RV leads implanted within two years of LVAD surgery appeared to be more stable as only the sensed amplitude was significantly affected. In contrast, three parameters were impacted in older leads. This is contrary to what would be expected, as older leads are generally considered to be more stable, and it does not have an obvious explanation. It can be hypothesized that lead-myocardium contact points with matured scarring may demonstrate less ability to remodel following post-implantation LV unloading with resultant septal shift and alterations in lead orientation. Perioperative lead changes stratified by lead age have not been assessed in other studies.

Mechanical disruption is also worth mentioning despite its infrequency because of the potential to cause irreversible damage and electrical failure of the ICD. This study revealed numerous types of complications that involved atrial, RV, and LV leads. However, while the impact of surgery was sometimes obvious, it was not always possible to determine if a lead dislodgement, for example, occurred secondary to surgery or if the surgery and associated workup identified changes in lead function which may have occurred due to lead evolution over time.

### Our data shares some, but not all, conclusions reached by previous studies

Our research shares much in common with prior studies but also offers new insights into the core question. Many previous investigations, ours included, found a significant decrease in the sensed amplitude threshold.^7^, ^8^, ^9^, ^10^, ^13^, ^14^ Capture threshold changes were variable in prior reports.^10^, ^14^ Only Black-Maier et al also investigated the high voltage impedance, though unlike our study, did not find a significant change.^13^ Moreover, no prior study reported either the pulse width or stratified data by age of the RV lead. Although Hu et al looked at similar outcomes, their approach was unique and thus cannot be easily compared to our study.^11^ Table 6 contains a summary of these studies.

**Table 6:** Comparisons to Previous Studies

Authors	Number of Patients Analyzed	% of RV Lead Dysfunction	Pre-LVAD Sensed Amplitude Threshold (mV)	Post-LVAD Sensed Amplitude Threshold (mV)	Pre-LVAD Pacing Impedance (Ω)	Post-LVAD Pacing Impedance (Ω)	Pre-LVAD Capture Threshold (V)	Post-LVAD Capture Threshold (V)	Pre-LVAD High Voltage Impedance(V)	Post-LVAD High Voltage Impedance(V)
Ambardekar, A et al	30	13.3% lead revision	9.2 (6.1−12.3)	5.7 (2.1−9.3)*	479 (361−597)	418 (324−512)*	4.3±6.7	11.0±16.8*		
Black-Maier, E et al	583	6.5% lead revision, of which 5.8% was not related to class I recall	12.5 (9.4−13.8)	4.0 (2.2−7.0)*	471 (419−523)	418 (353−492)	1.0 (0.75−1.1)	1.5 (1−2.7)*	49	41
Boudghène-Stambouli, F et al	23	0%	12 (8.0−15.9)	9 (8.1−12.4)*	520 (483−592)	456 (407−549)	0.75 (0.5−1.0)	0.95 (0.74−1.03)		
Foo, D et al	15	0%	10.9 (5.7−16.1)	7.2 (3.8−10.6)*	642 (422−882)	580 (368−792)*	0.8 (0.2−1.4)	1.4 (0.4−2.4)*		
Galand, V et al	122	0% revision, but up to 55% dysfunction	11.5 (7.9−13.8)	9.0 (6.3−12.0)*	490 (430−582)	445.5 (399−494)∗	0.75 (0.5−1)	0.75 (0.5−1)		
Thomas, I et al	44	1 lead fracture, 18% with ICD modifications	10.0 (7.6−13.6)	7.9 (5.9−9.3)*	440 (416−494)	404 (380−437)	0.82 (0.47−0.91)	1.14 (0.91−1.53)		

*Significant result in previous study

As for secondary outcomes, one of the more clinically relevant was direct impact of LVAD implantation on the RV lead. Rates of RV lead dysfunction requiring revision have ranged from 0–13.3% in prior studies. The analysis of RV lead dysfunction provided by previous analyses has been restricted by limited sample size.^7^, ^8^, ^10^, ^11^, ^14^ Between studies of similar population size to this article (Black-Maier et al, Galand et al), there were differing methods of reporting lead dysfunction.^9^, ^13^ Black-Maier et al recognized a clinically significant RV lead dysfunction rate of 5.8%, characterized as those requiring lead revision.^13^ Causes of lead revision were then classified as either increased/decreased impedance, failure to capture, increased capture threshold, oversensed amplitude, or undersensed amplitude. They utilized a similar set of criteria as Galand et al, including a >50% decrease in RV sensed amplitude, a >100 Ω increase/decrease in RV pacing impedance, or a >50% increase in RV pacing threshold.^13^ Galand et al reported an RV lead dysfunction rate of 55%, although none of the patients evaluated required lead revision.^9^ For the purposes of our study, we defined RV lead disruption as a post-operative change in a lead requiring either device reprogramming or lead revision.

### Limitations

Due to its retrospective nature, our study was limited first and foremost by the availability of complete ICD interrogations available for analysis. As 169 of the original 268 in the study population were excluded due to incomplete ICD data, selection and non-response bias may have contributed to these results. Moreover, although we limited our study to ICD interrogations recorded within one year pre- and post-LVAD implant, and used the interrogations taken closest to LVAD surgery, this allows for variation in timing. Some post-LVAD interrogations, for example, were taken the day of or the day after the surgery. Others were recorded in the weeks or months that followed. Because previous studies have reported that adverse changes in ICD parameters can improve over time,^11^ a study focusing on serial interrogations over a longer period of time may provide better insight. Applying this to our study, the effect on lead parameters that was discovered may represent a temporary aberration due to sequential proximity to LVAD implantation. Moreover, because complete ICD interrogations were part of the inclusion criteria, our study may have been biased towards patients whose ICDs malfunctioned in a manner serious enough to merit interrogation. Last, this study represents the experience of a single institution. It is possible that other institutions with dissimilar patient populations may have a different experience based on their best practices. The role of ICDs has been questioned in patients with LVADs, and therefore changes in RV lead function may be of limited clinical significance. This issue was not directly addressed by our study and is beyond the scope of this analysis.

## Conclusion

Several studies have now shown that LVAD implantation significantly affects ICD RV lead parameters. Our study demonstrates that these changes can be dependent on the age of the RV lead and suggests that the age of other device components may also be relevant. We have also shown that the high voltage impedance can be impacted in addition to the previously noted changes in the sensed amplitude and capture thresholds. Because of the demonstrated potential for ICD disruption, clinicians should strongly consider routine ICD interrogations in the peri-operative period and beyond to evaluate for changes that may herald electrical failure or lead disruption. Moreover, the potential for device-related complications from the surgery should be discussed with patients prior to progressing with LVAD placement.

## Funding

No funding was obtained to produce this work.

## Disclosures

The authors have nothing to disclose. There are no relationships with industry. There are no conflicts of interest. The authors did not use artificial intelligence to write this article.

## CRediT authorship contribution statement

All authors were involved in the conception and design of the project. All authors contributed meaningfully to the manuscript.

## Declaration of Competing Interest

The authors declare that they have no known competing financial interests or personal relationships that could have appeared to influence the work reported in this paper.
